# Feasibility of using Facebook for HIV prevention: Implications for translational research among justice-involved women who use drugs in rural Appalachia

**DOI:** 10.1017/cts.2022.497

**Published:** 2022-11-09

**Authors:** Michele Staton, Megan F. Dickson, Erika Pike, Sean Young

**Affiliations:** 1 Center on Drug and Alcohol Research, University of Kentucky, Lexington, KY, USA; 2 Department of Behavioral Science, University of Kentucky College of Medicine, 1100 Veterans Dr., Lexington, KY, USA; 3 University of California at Irvine, Institute for Prediction Technology, Irvine, CA, USA

**Keywords:** Justice-involved women, HIV prevention, rural health, social media, intervention

## Abstract

**Background:**

Justice-involved women from rural Appalachia face significant barriers to the utilization of evidence-based HIV prevention interventions in spite of high rates of injection drug use and risky sexual practices. Adapting evidence-based practices to incorporate the cultural uniqueness of the target population is needed in order to advance translational and clinical science in this area. This study provides a descriptive overview of indicators of feasibility and acceptability of an adapted version of the National Institute on Drug Abuse Standard HIV prevention intervention for delivery using Facebook through a small randomized controlled pilot study with rural Appalachian women.

**Method:**

Study methods include the random selection of rural Appalachian women from two local jails, screening for study eligibility, baseline data collection, random assignment to study interventions, and follow-up in the community three months post-release.

**Results:**

Results indicate that the feasibility of the approach was supported through study enrollment of the target population who reported regular Facebook use and HIV risk behaviors including drug use and sex. Acceptability of the intervention was demonstrated through enrollment in the study intervention, engagement in the intervention through Facebook, and indicators of HIV/HCV knowledge.

**Conclusions:**

Study findings contribute to the critical and unmet need to advance translational science on the delivery of evidence-based prevention interventions in real-world rural Appalachian settings to understudied, vulnerable individuals who are often overlooked in targeted prevention efforts.

## Introduction

The Appalachian region has the highest rates of morbidity, disability, and impaired quality of life in the nation [1]. Health disparities are attributed in large part to the opioid epidemic, which has significantly and disproportionately affected this region. These health disparities are compounded by a dearth of behavioral health treatment, which contributes to a lack of opportunities for related resources (such as human immunodeficiency virus (HIV) or hepatitis C virus (HCV) prevention) among this vulnerable population. Rural women in Appalachia bear greater drug-related health burdens due to limited access to treatment and prevention services (e.g.,[2–4]). Rural Appalachia has a number of health disparity indicators such as high rates of chronic illness, chronic depression, poor maternal health, and high rates of suicide [3,5], that signal a need for HIV prevention research among this group of women who are at heightened risk. In addition, rural Appalachian women face significant barriers to health services compared to their urban counterparts such as availability and access, as well as the fit between existing services and the cultural uniqueness of rural women [3,4]. Women who use drugs are also often stigmatized, discriminated against, or treated poorly in traditional health care settings [6,7]. Despite significant disparities in health and service utilization, empirical research on efforts to increase HIV prevention strategies among this disadvantaged group of Appalachian women has been largely overlooked.

Limited HIV prevention services are particularly problematic considering rural Appalachian women’s high-risk behaviors. In general, women are disproportionately vulnerable to contracting HIV due to unprotected heterosexual contact with risky partners, impaired condom use judgment, lack of agency for negotiating safer behaviors with partners, and being in violent and abusive relationships [7]. These vulnerabilities are compounded for rural Appalachian women [3,8] who are likely to report injection initiation due to pressure from peers and partners and to be injected by a partner, using the partner’s syringe, and in the partner’s home [9]. Another study found that rural Appalachian women report having more lifetime sexual partners, are more likely to have a partner who injected drugs, and are more likely to use drugs before sexual intercourse compared to non-injectors [3]. Women’s high-risk injection practices (sharing needles and other injection equipment) are not only associated with having a high-risk partner who injects but also with the perceptions of less power within that relationship [10], which may be associated with adherence to more traditional gender roles within relationships.

Studies of rural Appalachian women who use drugs can be challenging because recruitment of this high-risk population may be limited by the lack of formal treatment opportunities, travel distances to study sites, and the general protective nature of rural social networks [11,12]. The current study utilizes local rural jails as venues for screening and recruitment of hard-to-reach, high-risk women who use drugs, followed by targeted prevention efforts in the community post-release. Jails are different than prisons because individuals typically stay for a shorter period of time and often have limited access to health and behavioral health services. While rural jails can provide a critical opportunity for outreach to high-risk individuals who use drug and who may not otherwise be exposed to interventions [3], the majority of women return to rural communities where services, including HIV prevention, are very limited. Thus, it is critical to focus HIV prevention interventions during the high-risk period of community re-entry due to the potential for resuming pre-incarceration high-risk behaviors.

Despite progress in the past two decades, current women’s prevention interventions continue to have limited focus on the broader cultural and social context of sex and drug use for women [7]. Currently, of the 228 interventions listed in the Centers for Disease Control Compendium of Evidence-based Interventions and Best Practices for HIV prevention, only five are tailored specifically for women who use drugs [13], and none are uniquely tailored for rural Appalachian women despite considerable risk. One evidence-based HIV prevention intervention, the National Institute on Drug Abuse (NIDA) Standard, alone and in combination with motivational interviewing, was significantly associated with reductions in risky sexual practices and injection behaviors among rural Appalachian women during 3 months post-release from jail [3]. The NIDA Standard was developed through a large, multisite NIDA-funded cooperative agreement in the late 1980s (National AIDS Demonstration Research – NADR) and 1990s (Cooperative Agreement for AIDS Community-Based Outreach/Intervention Research), which were funded to educate high-risk injectors and encourage adoption of HIV prevention practices (e.g., [14]). NIDA Standard content focuses on reducing high-risk practices like needle use and cleaning strategies, safe sex practices including the latex condom, and the importance of drug treatment, and it has been successfully modified for women over the last two decades [15,16] with demonstrated efficacy.

Even with evidence-based HIV prevention interventions like the NIDA Standard, limited efforts have targeted intervention adaptation for new contexts and underserved, high-risk populations [17–19]. In essence, the feasibility and acceptability of HIV prevention interventions depend on targeted, high-risk individuals having access to those interventions. Social media applications like Facebook have grown in public health research in recent years (e.g., [20–22]) and have the potential to expand access to HIV prevention education, as demonstrated through research focused on hard-to-reach, high-risk individuals (e.g., [23,24]).

Among rural Appalachian women who use drugs, one study found that almost two-thirds of a randomly selected sample reported having a Facebook account that they checked regularly, and study retention and follow-up were significantly higher among Facebook users [25]. Thus, considering (1) the effectiveness of the NIDA Standard and (2) rural Appalachian women’s regular Facebook use, this evidence-based intervention was adapted for rural Appalachian women using the ADAPT-ITT framework during Phase 1 of this trial [26]. Through a series of focus groups with key stakeholders and theater tests [26], it was apparent that Facebook, rather than other social media platforms, was the most frequently used among rural women. The specific features of Facebook described as desirable by women included the range of content (silly memes to health care information), versatility for communication with family and friends, and a source of knowledge (ranging from health care information to where to buy drugs) [26]. Based on the comfort level with Facebook, specific intervention adaptations for this study included the development of online video demonstration of specific risk reduction activities (e.g., cleaning equipment) and *tailored* to women (e.g., how to put a condom on someone else) rather than simply reading the content from the NIDA Standard cue cards. Presenters in videos and postings were women from rural Appalachia who are familiar with the target population. Jargon and medical terminology were limited and replaced with layperson’s language at approximately a sixth-grade reading level and tailored with culturally appropriate language from feedback from study personnel. Posted intervention content remained available on the private Facebook group page continuously. Finally, local information about prevention resources for women (e.g., syringe exchange programs, health department) was regularly shared in the private group. As a follow-up to the adaptation process, the current study provides a descriptive overview of findings from a small randomized pilot trial to ascertain feasibility and acceptability of the adapted intervention with rural Appalachian women.

## Materials and Methods

### Sample Rationale and Study Participants

According to the seminal work by Leon and colleagues [27], pilot studies such as this do not generally require extensive sample size calculations, and it is recognized that “effects” generated from pilot studies cannot be utilized to power the eventual larger trial due to the wide confidence intervals often obtained in small pilots [27]. In the current study, the goal was not to conduct a fully-powered trial to detect significant differences in risk behavior based on the intervention over time; but rather a small feasibility pilot study to better understand intervention components as tested with the target population. Based on recommendations for sample size considerations in other pilot and feasibility studies (e.g., [28]), the sample size was based on the confidence intervals for number of participants needed to assess key aspects of intervention feasibility and acceptability. With a confidence interval width of 10%, the proposed sample size of 60 participants was sufficient to examine feasibility and acceptability of the NIDA Standard using Facebook (n = 30) compared to traditional NIDA Standard (n = 30) in this pilot trial.

Study participants (N = 60) were adult women (aged 18 and older) incarcerated in one of two targeted recruitment jail sites in rural Appalachia. Recruitment days were randomly selected at the beginning of each month to obtain a generalizable sample considering high turnover in the jail facilities. Women housed in each jail were then randomly selected and screened for study eligibility which included 1) moderate substance use risk based on the NIDA-modified Alcohol, Smoking, and Substance Involvement Screening Test (NM-ASSIST) score of 4+ for any drug; 2) self-reported sexual risk behavior in the 3 months before incarceration; 3) regular user of Facebook when living in the community; 4) self-reported HIV negative status; 5) residing in a designated Appalachian county before incarceration; and 6) willingness to participate.

### Measures

#### Demographics

Demographic variables included age (a continuous measure of self-reported age at the time of the baseline), education (a continuous measure of total number of years of formal education), race (self-reported racial group; categorically coded as White vs. non-White), marital status (categorically coded as married or living with someone as married vs. other), employment (percentage of women reporting any employment including full-time or part-time prior to incarceration vs. not working), and total income from all sources in the 3 months prior to incarceration. The county of recruitment was also coded as the location of the rural jail (Perry or Leslie) in order to assess potential site differences.

#### Intervention group

Participants were randomly assigned to one of two intervention conditions: 1) NIDA Standard alone (n = 30) or 2) Facebook (FB) NIDA Standard (n = 30).

#### Feasibility

Feasibility indicators included the following measures: (1) *Study enrollment* – the proportion of women agreeing to enroll in the study out of all possible participants approached as an indicator of “reach.” (2) *Study exclusion* – the proportion of women excluded due to not meeting study criteria. (3) *Intervention initiation* – the number of participants randomized to the FB NIDA Standard who accepted the friend request to join the general study Facebook page (offered to all study participants), as well as the invitation to join the private intervention site (offered only to the FB NIDA Standard group).

#### Acceptability

Indicators of acceptability included the following measures: (1) *Intervention engagement* – the number of FB NIDA Standard group participants who viewed the weekly intervention posts; (2) *Time of engagement* – the time between accepting the FB NIDA Standard group invitation and completion of the 3-month follow-up; (3) *Facebook interaction* – active participation in the intervention through responding to wall postings, “likes,” “shares,” and other contributions to the discussion; (4) *HIV/HCV knowledge* – sum of correct responses on scales measuring knowledge associated with HIV and HCV (higher scores indicate greater knowledge).

### Procedure

**Fig. 1.**
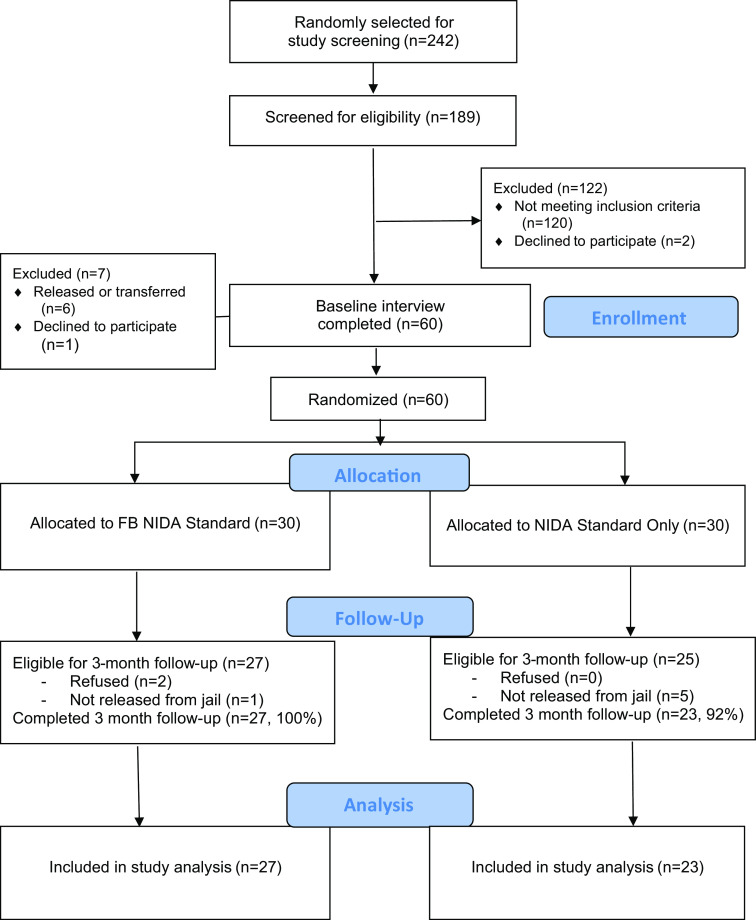
Study CONSORT. NIDA = National Institute on Drug Abuse.

This feasibility pilot study used simple random sampling to recruit hard-to-reach, rural Appalachian women who use drugs from two jails. A detailed description of research procedures, including random selection, is described in detail elsewhere [29], and program enrollment is described in the study CONSORT (see Fig. 1). Potential participants were randomly selected from daily jail census reports, and all women with an anticipated release date in the next three months who were on site on the screening day had an equal chance of being selected for screening. Potential participants were screened for study eligibility via interview while they were incarcerated (either face-to-face or via Zoom videoconferencing following implementation of COVID-19 restrictions). Participants were paid $25 for the baseline interview data collection only, not participation in the intervention. All study procedures were approved by the university IRB and protected under a federal Certificate of Confidentiality.

During the baseline session, research staff completed a one-on-one data collection interview with participants, followed by HIV/HCV pre-test counseling using cue cards from the NIDA Standard to provide education on the risks associated with contracting HIV and HCV through drug injection and risky sexual practices. Study research staff were trained and certified as HIV/HCV counselors by the State Department of Public Health. All participants were given the opportunity to be HIV and HCV tested using the OraQUICK *ADVANCE®* Rapid HIV-1/2 and OraQUICK *ADVANCE®* Rapid HCV Antibody Test kits, which have demonstrated sensitivity and specificity. Baseline and testing procedures took place in a private office in each jail, and test results were available within 20 min. Participants also received post-test counseling which included an explanation of the test result, referrals to resources either in the jail or in the community, and information about how to remain safe in the community. In summary, all study participants received two sessions of the NIDA Standard which included 1) pre-test counseling and 2) post-test counseling. Following the baseline and testing/counseling procedures, participants were randomly assigned to intervention condition using Research Randomizer (www.randomizer.org).

During the informed consent, participants were informed about the intervention random assignment and that they had a 50/50 chance of being assigned to either of the intervention conditions. Participants were either assigned to the 1) NIDA Standard alone or 2) Facebook (FB) NIDA Standard for 12 weeks post-release from jail. Participants’ release dates from the jail were monitored by research study staff. After release, all study participants received a friend request from the general Facebook study site monitor. After accepting the friend request, participants in the FB NIDA Standard condition were then sent an invitation to join the FB NIDA Standard intervention group. Participants in the NIDA Standard group alone only received the two initial sessions in jails (pre-test counseling and post-test counseling) and did not receive any additional contacts from the monitor until the 3-month follow-up.

Participants in the FB NIDA Standard group had the opportunity to participate in up to 12 weeks of the study intervention following their release from jail. Research staff discussed the confidentiality parameters of the “private” Facebook group (generically referred to as “Women’s Health Study”) during the informed consent process. Participants in this condition received a welcome video explaining the intervention and three introductory videos during week one (basic information about HIV, HBV, and HCV) followed by weekly wall posts by research study staff on their newsfeed with adapted and tailored content from NIDA Standard. Any interaction by the participants in the private intervention group did not appear in their newsfeed in order to maintain their confidentiality. One time each week, videos of NIDA Standard content were posted to the page or previous posts of that video were “bumped” by adding a comment to the video post. Videos were posted and “bumped” in the order the content would be delivered in the NIDA Standard. Participant interactions in the Facebook study group (e.g., video views, post likes) were monitored and recorded daily by the site monitor. Participants could have viewed up to 14 total videos, including 3 introductory videos during week one, followed by one video per week for the remaining 11 weeks.

All participants in both study conditions were followed 3 months post-release in the community. As shown in Fig. 1, of the 60 participants who completed a baseline interview, 30 participants were randomly assigned to each study condition: 1) NIDA Standard only, or 2) Facebook NIDA Standard. A total of 6 women (5 participants in the NIDA Standard only, 1 participant in the FB NIDA Standard condition) were not released from jail. In addition, 2 refused to complete a follow-up interview (both in the FB NIDA Standard condition). Of the 52 remaining eligible participants, 3-month follow-up interviews were completed with 50 (96.2% follow-up rate). Follow-up rates did not significantly differ by intervention condition with 27 follow-ups completed in the FB NIDA Standard group and 23 in the NIDA Standard alone condition. Participant locating and tracking methods included phone calls, flyer mailings, internet searches, and regular contact on Facebook. The 3-month follow-up interview was conducted either face-to-face or via phone or videoconferencing (depending on the participant’s comfort level under COVID restrictions), and participants were paid $25 for the follow-up data collection (not participation in the intervention), as well as a $25 bonus for completion of all research activities. The incentives are consistent with other studies at our Center with similar research participant populations and approved by the state Department of Corrections and university IRB.

### Analytic Plan

First, all study participants who were released to the community and received a friend request from the general Facebook study page were profiled using descriptive statistics (n = 54). Specifically, the sample’s demographic characteristics, acceptance of the friend request to join the general Facebook study page, and acceptance of the invitation to join the FB NIDA Standard intervention page (if applicable) were included. Next, a series of chi-square tests and *t*-tests were used to compare participants who were randomized to the NIDA Standard alone group (n = 25) to those individuals randomized to the FB NIDA Standard group (n = 29) on indicators of feasibility and acceptability such as intervention interaction and engagement. Finally, bivariate analyses were used to identify differences across groups in HIV and HCV knowledge which included only the sample of individuals who completed both the baseline and 3-month follow-up (N = 50), including a paired sample t-test comparing scores at baseline to scores at follow-up for each of the intervention groups. Analyses were conducted using IBM SPSS version 27.0.

## Results

### Demographics

Study participants were white (100%), with an average age of 36.6 years. The majority had children (92.6%), and 16.7% reported being married or living as married at baseline. On average, participants had completed 12.2 years of education. Only 7.4% reported being employed at least part-time in the three months before incarceration with an average income of $6,656. There were no significant differences in any of the study measures based on recruitment site, follow-up status (completed vs. not completed), or across intervention groups.

### Feasibility

Feasibility was assessed using a number of indicators, including *study enrollment*. Study enrollment included the proportion of women agreeing to participate in the study out of all possible women selected for screening. As shown in Fig. 1, during the enrollment period, 242 women were randomly selected from the two target jails and 189 (78%) participated in the study screening sessions. Only 23 refused to participate at the time of study screening (10%). Other reasons for not screening included being released early (n = 7), being out of the facility on screening day (n = 17), and other reasons (n = 6).

*Study exclusion* was also assessed as an indicator of feasibility. Of the 189 who participated in study screening, 67 (35%) met study inclusion criteria. The majority of those who were excluded (n = 91) did not have a release date in the upcoming 3 months. Few participants were excluded for not being a regular Facebook user (n = 10), not engaging in risky sexual practices (n = 14), not meeting the substance use criteria (n = 6), and not being from an Appalachian county (n = 23). Of the 67 who met study inclusion criteria, 60 agreed to enroll in the study (7 did not enroll due to being released or transferred [n = 6] and refusing to participate [n = 1])

*Intervention initiation* included two steps. First, everyone in the study who was released from jail was sent a friend request to the general Facebook study site. Of the 54 participants who were released to the community and sent a request to the general Facebook study site, 15 of 29 (51.7%) in the FB NIDA Standard group and 15 of 25 (60.0%) participants in the NIDA Standard alone group accepted the friend request within the targeted 3-month study intervention timeframe. Second, those in the FB NIDA Standard group who accepted the friend request for the general study site were sent a separate invitation to join the private intervention group. Of the 15 participants in this condition who joined the general Facebook study site, all 15 were invited to join the private FB NIDA Standard intervention page and 9 individuals accepted the intervention group invitation (60.0%).

### Acceptability

Acceptability was assessed through a number of indicators including *intervention engagement,* which was defined as the number of participants in FB NIDA Standard group who viewed the weekly intervention posts. Of the 9 participants who accepted the invitation to join the private FB NIDA Standard group, 100% viewed at least one session of the intervention, with an average of 6.9 videos viewed out of a possible 14 (SD 2.3, range 4–11). The average number of days to accept the invitation was 5.2 (SD 6.7, range 0–20), suggesting that participants who were engaged in the intervention did so relatively early. Of the 9 individuals engaged in the intervention site, 8 (89%) completed a 3-month follow-up, with an average of about 125 days (SD 80.8, range 36–251) of intervention participation.

In addition to intervention engagement, acceptability was also measured through *Facebook interaction* and the extent to which participants responded to intervention content through wall postings, “likes,” “shares,” and other contributions to the discussion. As shown in Table 1, nearly three-quarters (72.2%) of participants interacted with the research team by sending them at least one message – 76.0% of those in the NIDA Standard alone group and 69.0% of those in the FB NIDA Standard group. It should be noted that three participants in the NIDA Standard condition sent a high number of messages, increasing the study mean. Participants from the full sample sent an average of 12.6 messages (SD 15.3; range 0–58). A number of participants from both groups also interacted with the research team by reacting to friend requests or messages.

**Table 1.** Facebook interaction by study participants by intervention group

	NIDA Standard only (n = 25)	Facebook NIDA Standard (n = 29)	Total (n = 54)
Percent who interacted with research team by sending Facebook messages	76.0%	69.0%	72.2%
Average # of messages sent by participant	16.6 (18.7)	9.1 (10.8)	12.6 (15.3)
Percent who reacted to a Facebook friend request or message from the research team	32.0%	24.1%	27.8%
Average # of reactions received from participant	1.1 (2.0)	0.6 (1.5)	0.8 (1.7)

NIDA = National Institute on Drug Abuse.

Finally, acceptability was measured through *HIV/HCV knowledge* regarding risks associated with certain drug use sharing practices and risky sexual behaviors. As shown in Table 2, participants in both conditions reported a fairly high degree of knowledge of HIV and HCV risk behaviors at baseline, but did not differ statistically across groups. HIV and HCV knowledge improved at follow-up in both conditions. Specifically, there were significant increases from baseline to follow-up in HIV knowledge for the NIDA Standard alone group (*t*(22) = −2.66, *p* = .014) and significant increases in HCV knowledge for both the NIDA Standard alone group (*t*(22) = −4.87, *p* < .001) and the FB NIDA Standard group (*t*(26) = −2.75, *p* = .011).

**Table 2.** HIV and HCV knowledge at baseline and 3-month follow-up by intervention group

	NIDA Standard only (n = 23)	Facebook NIDA Standard (n = 27)	Total (n = 50)
**HIV Knowledge Scale (range 0–18)**			
Baseline	16.9 (1.2)	16.9 (1.2)	16.9 (1.2)
Follow-up	17.7 (0.5)^+^	17.5 (1.1)	17.6 (0.9)^++^
**HCV Knowledge Scale (range 0–9)**			
Baseline	7.7 (0.8)	7.9 (0.7)	7.8 (0.7)
Follow-up	8.6 (0.7)^+++^	8.4 (0.8)^+^	8.5 (0.8)^+++^

Note: Significance noted at ^+^ p≤.05; ^++^ p≤.01; ^+++^ p≤.001; between baseline and follow-up. HCV = hepatitis C virus; HIV = human immunodeficiency virus; NIDA = National Institute on Drug Abuse.

## Discussion

Despite engaging in high rates of HIV risk behaviors associated with drug use and unprotected sex, HIV prevention interventions are limited for rural Appalachian women. Through utilization of the ADAPT-ITT framework during Phase 1, our team modified the evidence-based NIDA Standard HIV prevention intervention for rural Appalachian women to address this critical gap in the literature. Specific adaptations were focused on content (delivery using videos by rural Appalachian women, targeted content to local resources), as well as context (delivery using Facebook) [26]. It is important to note that during our Phase 1 developmental work, study participants identified Facebook as their primary form of social media. It is important for future research to use a similar strategy among other populations to ensure that interventions are delivered using the ideal form of social media used by the targeted, intended population. The current study provides a descriptive overview of indicators of feasibility and acceptability of the adapted intervention through a small randomized pilot test with 60 rural Appalachian women recruited from local jails.

Study indicators of feasibility were measured through study enrollment and intervention initiation, which is consistent with other work on adapted interventions [30,31]. Findings related to study enrollment, including exclusion criteria, suggest that among a randomly selected sample of 189 women who participated in screening, study methods were highly feasible in reaching the proposed sample of the target population. The majority of women were screened out due to not being released from jail rather than not meeting HIV risk behavior criteria or regular Facebook use. In fact, of the 189 women screened, very few did not meet study criteria associated with Facebook use (n = 10), engaging in risky sex (n = 14), and not meeting substance use criteria (n = 6). These findings are consistent with other studies (e.g., [3,25]) which suggest that local jails provide ideal venues to outreach and recruit high-risk women who may otherwise not be engaged in treatment or prevention resources.

Engagement in Facebook generally and specific engagement in the FB NIDA Standard intervention were also assessed as indicators of feasibility and acceptability. Findings indicated that more than half of women released from jail accepted the friend request to join the general Facebook study site. Of those randomly assigned to the FB NIDA Standard group, 60% also accepted the invitation to join the private intervention group. While overall slightly lower than other Facebook intervention groups [30], it is important to remember that the study enrollment took place in jail, and engagement in the Facebook site was not initiated until after release. Consistent with other studies on this high-risk population, the timing of release from jail and community re-entry can be characterized by a number of chaotic events [32]. Among those who joined the Facebook study site and intervention group, engagement was demonstrated through messages with the research team, responses to the team, and watching study intervention group videos. While each of these are important indicators of intervention acceptability, it is important to note that this is the first trial using the NIDA Standard content through the Facebook platform, and the finding that women viewed nearly 7 videos is important in future trials considering a dose threshold as it relates to intervention effectiveness. In addition, future research should include more targeted strategies to proactively connect women to the Facebook intervention site, particularly for those who are incarcerated and preparing for community release. In addition, future studies should consider qualitative research to better understand barriers to intervention engagement using Facebook platforms.

Finally, acceptability was measured through HIV/HCV knowledge regarding risks associated with certain drug use sharing practices and risky sexual behaviors. Other studies with justice-involved rural women in Appalachia have suggested that HIV and HCV knowledge is associated with reductions in HIV risk behaviors, particularly drug use [33]. Findings from this study indicate that participants in both intervention conditions reported a fairly high degree of knowledge of HIV and HCV risk behaviors at baseline, which improved significantly at follow-up in both conditions, but did not differ statistically between intervention groups. The lack of differences between groups is likely attributable to all intervention content being derived from the NIDA Standard, which is the same in both conditions and only varied based on the delivery platform and frequency of session content. Understanding the extent of knowledge about HIV and HCV risks is critical in the delivery of prevention interventions because there can be a conceptual gap between *knowledge* of a risk and implementation of strategies to *change* risk behaviors [34,35]. Future research should focus on the unique challenges that may exist between knowledge of risk and risk behavior change for rural Appalachian women.

HIV prevention is understudied in rural Appalachia because, in general, HIV prevalence rates are considerably lower than the rest of the nation. One study reported that the HIV prevalence is 57% lower in the Appalachian Region than in the nation as a whole [36]. While the overall prevalence of HIV may be generally lower, rural areas of Appalachia have been identified as being at heightened risk for an HIV outbreak [37], largely attributed to the combination of high rates of poverty, low access to health and behavioral health care, and rampant injection drug use. Considering the cultural uniqueness of rural Appalachia, delivery of evidence-based HIV prevention interventions that have been developed and tested with other high-risk groups may not be effective in the absence of targeted adaptation for content and context. Adaptations for rural women in particular must incorporate concepts that underlie unique risks and vulnerabilities for women. These basic assumptions [38], tailored for rural Appalachian women [32], include the following: a) social status is a central feature in understanding risk; for rural Appalachian women, most live in extreme conditions of poverty, and decision-making regarding risk behavior is often influenced by her ability to survive; b) women’s identity is closely tied to connections with others; the fear of disconnection or abandonment influences decision-making; c) male partners are key players in women’s risk; among rural Appalachian women, decisions about injection sharing practices are closely tied to high-risk partners; and d) experiences of victimization and violence serve as barriers to risk reduction; the cultural context for victimization and protection of abusive partners is different in Appalachia.

This study has some notable limitations. The study incorporated random selection for the small pilot to examine social media use and HIV/HCV risk behavior from two jails in rural Appalachian. While findings indicate study methods facilitated reaching the intended target population for the HIV prevention intervention, results should be interpreted with limited generalizability to other substance-using women involved in the criminal justice system outside of rural Appalachia. Also, Facebook was selected as the platform for this intervention because it was the most commonly used form of social media among this high-risk group of rural women. Therefore, findings related to feasibility and acceptability may not translate to other forms of social media. The focus of this analysis was on indicators of feasibility and acceptability, but the small sample size in general, coupled with further reductions based on those who accepted the initiation to join the FB NIDA Standard, limited more complex analysis related to individual-level factors which may impact intervention effectiveness such as mental health or victimization history. Efforts to increase connection to the Facebook or other social media sites should be a focus of future research. It should also be noted that intervention content related to high-risk drug use and sexual behavior may not have been as relevant for women leaving jail and refraining from high-risk practices, which may have also played a role in intervention initiation and engagement. Considering that all study participants were incarcerated at the time of the baseline and subsequently released from jail, there may always be concerns related to confidentiality. Participants were assured of IRB protections, as well as the protections of the Certificate of Confidentiality at baseline and at the 3-month follow-up. Finally, while preliminary analysis did not detect any notable differences in data collected in person versus videoconferencing, it is always possible that the onset of COVID-19 and resulting restrictions may have impacted participants’ study involvement over time.

Despite these limitations, understanding the feasibility and acceptability of adapted interventions for hard-to-reach women at high risk for HIV has important implications for translational science. Considering the importance of reaching this high-risk population in jails, establishing partnerships between research teams and criminal justice partners is critical for the success of study implementation. Future research must continue to examine the cultural uniqueness of target populations (both in terms of content and context) in the innovative adaptations of evidence-based practices. While this small-scale feasibility trial focused on the use of Facebook as an intervention delivery platform, future research should examine the long-term impact of intervention content on reduction of HIV/HCV risk behaviors among Appalachian women. While beyond the scope of this paper, it is possible that perceptions of intervention acceptability may have an impact on study outcomes, which should be examined in future research. There is a critical need to advance knowledge and research on the delivery of evidence-based prevention interventions in real-world rural Appalachian settings to high-risk women who are often overlooked in targeted prevention. Future research should also examine real-world settings in rural communities to reach high-risk women for prevention efforts other than criminal justice venues. This study has relevance for translational science by describing critical elements of feasibility and acceptability in the innovative delivery of a relatively low-cost, translatable, social media-based HIV prevention intervention for high-risk, understudied, and vulnerable rural Appalachian women during a time of significant public health risk.

## References

[1] Appalachian Regional Commission (ARC). *County Economic Status in Appalachia, FY 2020*. 2020. (https://www.arc.gov/map/county-economic-status-in-appalachia-fy-2020/)

[2] Substance Abuse and Mental Health Services Administration, Center for Behavioral Health Statistics and Quality (SAMHSA). *The TEDS Report: A Comparison of Rural and Urban Substance Abuse Treatment Admissions*. 2012. (http://www.samhsa.gov/sites/default/files/teds-short-report043-urban-rural-admissions-2012.pdf)

[3] Staton M , Ciciurkaite G , Oser C , et al. Drug use and incarceration among rural Appalachian women: Findings from a jail sample. Substance Use & Misuse 2018; 53(6): 931–941. 29161158 10.1080/10826084.2017.1385631 PMC6121714

[4] Statz M , Evers K . Spatial barriers as moral failings: What rural distance can teach us about women’s health and medical mistrust author names and affiliations. Health & Place 2020; 64: 102396. doi: 10.1016/j.healthplace.2020.102396 32739783 PMC7391386

[5] American College of Obstetricians and Gynecologists (ACOG). Committee Opinion No. 586: Health disparities in rural women. Obstetrics & Gynecology 2014; 123(2): 384–388. 24451676 10.1097/01.AOG.0000443278.06393.d6

[6] Surratt HL , Otachi JK , McLouth CJ , Vundi N . Healthcare stigma and HIV risk among rural people who inject drugs. Drug and Alcohol Dependence 2021; 226: 108878. doi: 10.1016/j.drugalcdep.2021.108878 34214880 PMC8355211

[7] Wechsberg WM , Deren S , Myers B , et al. Gender-specific HIV prevention interventions for women who use alcohol and other drugs: The evolution of the science and future directions. JAIDS Journal of Acquired Immune Deficiency Syndromes 2015; 69(Supplement 2): S128–S139. doi: 10.1097/QAI.0000000000000627 25978479 PMC4505613

[8] Schalkoff CA , Lancaster KE , Gaynes BN , et al. The opioid and related drug epidemics in rural Appalachia: A systematic review of populations affected, risk factors, and infectious diseases. Substance Abuse 2020; 41(1): 35–69. 31403903 10.1080/08897077.2019.1635555 PMC7012683

[9] Young AM , Larian N , Havens JR . Gender differences in circumstances surrounding first injection experience of rural injection drug users in the United States. Drug and Alcohol Dependence 2014; 134: 401–405. doi: 10.1016/j.drugalcdep.2013.10.013 24216393 PMC3874445

[10] Staton M , Strickland JC , Tillson M , Leukefeld C , Webster JM , Oser CB . Partner relationships and injection sharing practices among rural Appalachian women. Women’s Health Issues 2017; 27(6): 652–659. 28882550 10.1016/j.whi.2017.07.005 PMC5701655

[11] Friedman, P . Meeting the challenge of social service delivery in rural areas. Welfare Information Network 2003; 7(2). (http://www.financeproject.org/publications/meetingthechallengeIN.htm)

[12] Leach CR , Schoenberg NE , Hatcher J. Factors associated with participation in cancer prevention and control studies among rural Appalachian women. Family & Community Health 2011; 34(2): 119–125. 21378508 10.1097/FCH.0b013e31820de9bf PMC3086267

[13] Centers for Disease Control (CDC). *Women and HIV: HIV Diagnoses*. 2021. (https://www.cdc.gov/hiv/group/gender/women/diagnoses.html)

[14] Coyle SL , Needle RH , Normand J . Outreach-based HIV prevention for injecting drug users: a review of published outcome data. Public Health Reports 1998; 113 Suppl 1: 19–30. 9722807 PMC1307724

[15] Surratt HL , Inciardi JA . An effective HIV risk-reduction protocol for drug-using female sex workers. Journal of Prevention & Intervention in the Community 2010; 38(2): 118–131. 20391059 10.1080/10852351003640732 PMC2879022

[16] Wechsberg WM , Lam WKK , Zule WA , Bobashev G . Efficacy of a woman-focused intervention to reduce HIV risk and increase self-sufficiency among African American crack abusers. American Journal of Public Health 2004; 94(7): 1165–1173. 15226138 10.2105/ajph.94.7.1165 PMC1448416

[17] McKleroy VS , Galbraith JS , Cummings B , et al. Adapting evidence–based behavioral interventions for new settings and target populations. AIDS Education and Prevention 2006; 18(supp): 59–73. doi: 10.1521/aeap.2006.18.supp.59 16987089

[18] Wenzel SL , Cederbaum JA , Song A , et al. Pilot test of an adapted, evidence-based HIV sexual risk reduction intervention for homeless women. Prevention Science 2016; 17(1): 112–121. 26103921 10.1007/s11121-015-0575-6

[19] Wingood GM , DiClemente RJ . The ADAPT-ITT Model: A novel method of adapting evidence-based HIV interventions. JAIDS Journal of Acquired Immune Deficiency Syndromes 2008; 47(Supplement 1): S40–S46. doi: 10.1097/QAI.0b013e3181605df1 18301133

[20] Kim SJ , Marsch LA , Brunette MF , Dallery J . Harnessing Facebook for smoking reduction and cessation interventions: Facebook user engagement and social support predict smoking reduction. Journal of Medical Internet Research 2017; 19(5): e168. doi: 10.2196/jmir.6681 28536096 PMC5461420

[21] Napolitano MA , Whiteley JA , Mavredes MN , et al. Using social media to deliver weight loss programming to young adults: Design and rationale for the Healthy Body Healthy U (HBHU) trial. Contemporary Clinical Trials 2017; 60: 1–13. doi: 10.1016/j.cct.2017.06.007 28611007 PMC5845797

[22] Naslund JA , Aschbrenner KA , Marsch LA , McHugo GJ , Bartels SJ . Facebook for supporting a lifestyle intervention for people with Major Depressive Disorder, Bipolar Disorder, and Schizophrenia: An exploratory study. Psychiatric Quarterly 2018; 89(1): 81–94. 28470468 10.1007/s11126-017-9512-0 PMC5758428

[23] Young SD , Jaganath D . Feasibility of using social networking technologies for health research among men who have sex with men: A mixed methods study. American Journal of Men’s Health 2014; 8(1): 6–14. 10.1177/1557988313476878 PMC3879119 23407600

[24] Young SD , Swendeman D , Holloway IW , Reback CJ , Kao U . Use of technology to address substance use in the context of HIV: A systematic review. Current HIV/AIDS Reports 2015; 12(4): 462–471. 26475670 10.1007/s11904-015-0295-3 PMC4749410

[25] Dickson MF , Staton-Tindall M , Smith KE , Leukefeld C , Webster JM , Oser CB. A Facebook follow-up strategy for rural drug-using women: Facebook follow-up. The Journal of Rural Health 2017; 33(3): 250–256. 27467119 10.1111/jrh.12198 PMC5274631

[26] Studts CR , Tillson M , Pike E , Staton M . Adaptation of the NIDA Standard for delivery via Facebook with justice-involved women in rural Appalachia. Implementation Research and Practice 2021; 2: 263348952110141. doi: 10.1177/26334895211014123 PMC9978655 37089991

[27] Leon AC , Davis LL , Kraemer HC. The role and interpretation of pilot studies in clinical research. Journal of Psychiatric Research 2011; 45(5): 626–629. 21035130 10.1016/j.jpsychires.2010.10.008 PMC3081994

[28] Thabane L , Ma J , Chu R , et al. A tutorial on pilot studies: the what, why and how. BMC Medical Research Methodology 2010; 10(1): 1. 20053272 10.1186/1471-2288-10-1 PMC2824145

[29] Staton-Tindall M , Harp KLH , Minieri A , et al. An exploratory study of mental health and HIV risk behavior among drug-using rural women in jail. Psychiatric Rehabilitation Journal 2015; 38(1): 45–54. 25799305 10.1037/prj0000107 PMC4372151

[30] Young SD , Cumberland WG , Lee SJ , Jaganath D , Szekeres G , Coates T. Social networking technologies as an emerging tool for HIV prevention: A cluster randomized trial. Annals of Internal Medicine 2013; 159(5): 318. 24026317 10.7326/0003-4819-159-5-201309030-00005 PMC3879120

[31] Kurtz SP , Stall RD , Buttram ME , Surratt HL , Chen M . A randomized trial of a behavioral intervention for high risk substance-using MSM. AIDS and Behavior 2013; 17(9): 2914–2926. 23732957 10.1007/s10461-013-0531-z PMC3809331

[32] Staton M , Dickson MF , Tillson M , Webster JM , Leukefeld C . Staying out: Reentry protective factors among rural women offenders. Women & Criminal Justice 2019; 29(6): 368–384. 32855583 10.1080/08974454.2019.1613284 PMC7449142

[33] Peteet B , Staton M , Miller-Roenigk B , Carle A , Oser C. Rural incarcerated women: HIV/HCV knowledge and correlates of risky behavior. Health Education & Behavior 2018; 45(6): 977–986. 29627991 10.1177/1090198118763879 PMC11195302

[34] Künzler-Heule P , Fierz K , Schmidt AJ , et al. Response to a sexual risk reduction intervention provided in combination with hepatitis C treatment by HIV/HCV co-infected men who have sex with men: a reflexive thematic analysis. BMC Infectious Disease 2021; 21(1): 319. 10.1186/s12879-021-06003-z PMC8022541 33823783

[35] Dowse R , Barford K , Browne SH . Simple, illustrated medicines information improves ARV knowledge and patient self-efficacy in limited literacy South African HIV patients. AIDS Care 2014; 26(11): 1400–1406. 24975116 10.1080/09540121.2014.931559 PMC4124945

[36] Health Disparities in Appalachia. *Creating a Culture of Health in Appalachia: Disparities and Bright Spots*. 2021. (www.healthy-ky.org)

[37] Van Handel MM , Rose CE , Hallisey EJ , et al. County-level vulnerability assessment for rapid dissemination of HIV or HCV infections among persons who inject drugs, United States. JAIDS Journal of Acquired Immune Deficiency Syndromes 2016; 73(3): 323–331. 27763996 10.1097/QAI.0000000000001098 PMC5479631

[38] Amaro H . Love, sex, and power: Considering women’s realities in HIV prevention. American Psychologist 1995; 50(6): 437–447. 7598292 10.1037//0003-066x.50.6.437

